# Influence of Type of Modified Binder on Stiffness and Rutting Resistance of Low-Noise Asphalt Mixtures

**DOI:** 10.3390/ma14112884

**Published:** 2021-05-27

**Authors:** Raman Pakholak, Andrzej Plewa, Wladyslaw Gardziejczyk

**Affiliations:** Faculty of Civil Engineering and Environmental Sciences, Bialystok University of Technology, 15-351 Bialystok, Poland; a.plewa@pb.edu.pl (A.P.); w.gardziejczyk@pb.edu.pl (W.G.)

**Keywords:** stiffness modulus, low noise pavement, rubber granulate, modified bitumen, rutting resistance

## Abstract

Low-noise asphalt mixtures are characterized by increased air void content. Their more open structure contributes to faster degradation within the operating temperature range. For this reason, binder modification is used in their production. The correct selection of modifiers allows one to significantly improve the technical properties of the mixtures. The article presents the results of tests of six types of mixtures: stone mastic asphalt (SMA8), porous asphalt (PA8), stone mastic asphalt reducing tire/road noise (SMA8 LA) and stone mastic asphalt reducing tire/road noise, with 10%, 20% and 30% content of rubber granulate (RG). Bitumen 50/70 modified with copolymer styrene butadiene styrene (SBS) and crumb rubber (CR) was used for the production of the mixtures. In order to determine the differences in the technical properties of the mixtures, the following parameters were tested: stiffness modules by indirect tensile testing of cylindrical specimens (IT-CY) in a wide range of positive temperatures, and resistance to permanent deformation using the British and Belgian methods with the use of double wheel tracker (DWT). The test results and their analysis confirmed that there was a significant improvement in the IT-CY stiffness modules of SBS and CR modified mixtures. Replacing more than 20% of coarse aggregate with RG causes a significant decrease in the stiffness of the mixture (by 90% in relation to the reference mixture SMA8 LA). The SMA mixtures obtained lower values of rutting resistance parameters (WTS and PRD) in water (Belgian method) compared to the results obtained in the air tests (British method). On the other hand, mixtures of PA, thanks to the compression of stresses in pores filled with water, obtained better results when the rutting resistance test was performed in the water (Belgian method).

## 1. Introduction

Development of the communication infrastructure and the increase in road traffic volume have resulted in an increase in the noise level generated by motor vehicles in the road surroundings [1]. “Quiet” road surfaces, which include PA, thin layers of BBTM type (French: Bétons Bitumineux Très Minces) with a maximum grain size 8 mm and layers of grit mastic SMA LA, reduce the level of tire/surface noise even up to 5–6 dB compared to asphalt concrete (AC) or SMA layers with a maximum aggregate grain size 11 mm [2]. However, the greater air void content in such mixtures (in the range of 10–20%) contributes to faster destruction of such surfaces compared to the standard solutions of surface layers [3]. The increased content of air voids requires the use of high-quality modified bitumen, which significantly determines the durability of the pavement in operating conditions. Positive results are obtained with the use of binders modified with the addition of SBS copolymer (e.g., Kraton 1192), CR from used car tires and a combination of these two modifiers [4,5,6]. Modifiers of this type contribute to a greater range of viscoelasticity, increasing the softening temperature, improving the resistance to technological and service aging, and increasing low temperature cracking resistance [7,8].

The formation of ruts is one of the most types of common damage to asphalt surfaces [9,10]. This process depends primarily on the physical and mechanical properties of the used asphalt mixtures, the materials used in the pavement structure, climatic conditions, load characteristics and road traffic volume [5,11,12,13]. The resulting permanent deformations prevent the proper drainage of water from the road surface, which significantly affects road safety and reduces driving comfort. Therefore, many research centers are looking for effective methods of counteracting the initiation of this type of damage [14,15,16,17]. The resistance of asphalt mixtures designed by the Superpave method with the use of a gyratory compactor and AC depends on the effectiveness of aggregate wedging [18,19]. The authors prove that the anti-rutting additive (ARA) and SBS-modified bitumen improve the rutting resistance of AC and SMA mixtures. On the other hand, in the case of mixtures with higher air void content and higher binder content, the rutting resistance is determined not only by the wedging of aggregate but also by the binder flexibility that connects the aggregate grains.

Laboratory tests of stiffness modulus and rutting resistance well characterize asphalt mixtures at positive operating temperatures, and at the same time allow one to assess the quality of the bitumen binders [20,21]. The modulus of asphalt mixture depends on temperature and is a key parameter determining the fatigue life of road pavement [22]. As the temperature drops, its value increases, making the layer stiffer but at the same time more brittle and prone to cracking. On the other hand, at high summer temperatures, the opposite phenomenon occurs—the stiffness modulus decreases and the resistance to deformations of the upper pavement layer decreases. There are many methods for testing the stiffness modulus of asphalt mixtures. Stiffness modulus as a function of temperature is most commonly tested using the four-point bending test on prismatic specimens (4PB-PR), indirect tension to cylindrical specimens (IT-CY) and cyclic indirect tensile test (CIT-CY) [23].

An important aspect is the high correlation of laboratory test results with the behavior of the pavement in “in situ” conditions when designing road pavement structures. Therefore, methods that test materials in devices imitating real operating conditions are being increasingly used. It was found that the widely used Hamburg Wheel Tracking Tester (HWTT) is suitable for generating parameters describing the plastic deformation of asphalt mixtures. The coefficient of variation (CoV) for all assessed laboratory rutting parameters was less than 30%, which proves the high repeatability of these parameters in relation to the results obtained in “in situ” conditions [24]. Two groups of laboratory methods are most often used to assess the rutting resistance of asphalt mixtures. The first group includes methods of testing a single layer [25,26,27], while the second group of methods enables the testing of a set of pavement construction layers [28,29,30].

On the basis of rutting resistance tests carried out in many research centers, it was found that the content, type and quality of the binder used for asphalt mixtures production have a significant impact on their rutting resistance [24,25,26,27]. The literature describes many methods of binder modifications, including polymers, hydrated lime and basalt fibers, polyalphaolefins, CR, synthetic wax and cellulose fibers [31,32,33,34,35].

The aim of the presented research to show how the modification of bitumen and the addition of RG change the stiffness modulus and rutting resistance according to the British and the Belgian methods of low-noise asphalt mixtures.

## 2. Materials

The test results for bitumen binders and mineral aggregates used for asphalt mixtures are described in publications [7,8], and selected technical properties of modified binders are presented in Table 1. The used copolymer Kraton 1192 contains 30% of styrene, and its molecular weight is 1.38 × 105 g/mol. CR used for modification, with a grain size of 0/0.8 mm, came from used car tires. The bitumen modification process consisted of heating the bitumen 50/70 to the temperature of 180 °C ± 5 °C, then adding 5% SBS copolymer or 10% CR or combined 2% SBS copolymer with 10% CR.

Bitumen binders were subjected to the following tests: penetration (at temperatures: 5 °C, 15 °C and 25 °C), softening point according to ring and ball method, Fraass Breaking Point (T_Fraass_,), dynamic viscosity (at temperatures: 90 °C, 110 °C and 135 °C), strain energy with the determination of the maximum tensile force (at temperatures: 5 °C, 15 °C and 25 °C) and elastic recovery (at temperatures: 15 °C and 25 °C). Laboratory tests were carried out for bitumen before and after the technological aging process. The simulation of the technological aging process in laboratory conditions was performed using the rolling thin film oven test (RTFOT) method according to the standard [36]. RG with grain size 1/4 mm was added to asphalt mixtures using the “dry” method in amounts of 10%, 20% and 30% by volume of aggregate, replacing the appropriate part of the mineral aggregate.

The tests were carried out on the following mixtures: stone mastic asphalt (SMA8), stone mastic asphalt reducing tire/road noise (SMA8 LA), porous asphalt (PA8) and stone mastic asphalt reducing tire/road noise, with 10%, 20% and 30% rubber granulate (SMA8 LA (10% RG), SMA8 LA (20% RG), SMA8 LA (30% RG)).

**Figure 1:**
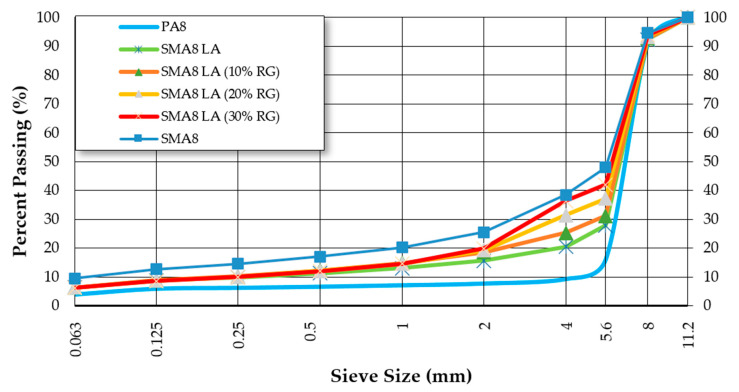
The particle size distribution of tested mixtures.

Cylindrical specimens (ϕ = 101.6 mm, h = 63.5 ± 2.5 mm) for testing the stiffness modulus IT-CY were compacted in accordance with the standard [37]. The samples for rutting tests were compacted in accordance with the standard [38] (300 mm × 400 mm × 40 mm plates). The particle size distribution of individual mixtures is shown in Figure 1. The binder content, air void content and bulk density of mixtures are presented in Table 2.

## 3. Experimental Methods

### 3.1. Indirect Tension to Cylindrical Specimens (IT-CY)

Elastic stiffness modulus determined in indirect tension (IT-CY) test according to the standard [23] is an important parameter that allows one to predict the behavior of mixture at positive operating temperatures at which its stiffness modulus decreases. Therefore, the IT-CY test was conducted in the following temperatures: 5 °C, 15 °C, 25 °C and 35 °C. The highest value of 35 °C was determined by the authors as the temperature at which it was possible to conduct a full set of tests of the mixtures. Too high deformation of samples containing mixtures with the addition of RG above this temperature was recorded. A controlled stress test was performed during the measurement. Dynamic load was applied five times to the sample vertically along the diameter. The time of force increase, measured from the moment of applying the force (zero value) to the maximum value, was 0.124 s. The maximum force generated a horizontal displacement of the sample equal to 5 μm. There was a 3-s delay between each force pulse. The test result was calculated automatically by the control program as the arithmetic mean of the stiffness modules for each of the five force pulse measurements. After the test, the sample was rotated 90° about the horizontal axis and tested again. If the average test result was within ±10% of the modulus value tested in the previous position, the stiffness modulus of the sample was calculated as the average of two measurements. If the measurement results did not fall within the acceptable range, they were not taken into account for further analysis. Duplicate test results carried out on the entire series of samples were within the limits of standard deviations. The final result was the arithmetic mean of 5 tested samples.

For the assumed load area coefficient equal to 0.6, the value of the stiffness modulus was determined from the following equations:

(1) $$S_{m}=\frac{F\cdot \left(\nu +0.27\right)}{z\cdot h}$$

(2) $$\nu =3.59\frac{z}{\Delta V}-0.27$$

where

F—the maximum force applied to the sample (N);
*z*—the amplitude of the horizontal displacement of the sample during loading (mm);
h—sample height (mm);
ν—Poisson’s ratio;
Δ*V*—maximum vertical displacement of the sample (mm).

### 3.2. Rutting Resistance Test Using the British and Belgian Method

The permanent deformation resistance test according to [39] was carried out in the DWT. This test is used to determine the deformability of asphalt mixtures as a result of repeated passage of the loaded wheel through the sample. The plates (height 40 mm, width 300 mm and length 400 mm) were compacted in an electromechanical plate compactor. Loading arms equipped with test wheels (203 mm × 50 mm) performed a reciprocating movement with a total wheel travel length of 230 mm. The test speed was 20 cycles per minute (40 wheel passes), the conditioning time was 4 h and the test temperature was 60 °C. Rutting resistance test was carried out in air according to the British method, while according to the Belgian method—samples were completely immersed in water during the test. Wheel tracking slope (WTS) was calculated based on the Equation (3):

(3a) $$WTS=\frac{d_{i}-d_{i/2}}{m},$$

where $d_{i}$ and $d_{i/2}$—rut depth after i and i/2 load cycles (mm);

(3b) m=(i−i/2)/1000,

Percentage of rut depth (PRD) after N cycles of the loading was calculated from the Equation (4):

(4) $$PRD=\frac{h_{2}}{h_{1}}\cdot 100\%,$$

where

*h*_1_—the initial rut depth (mm);
*h*_2_—the final rut depth (mm).

**Figure 2:**
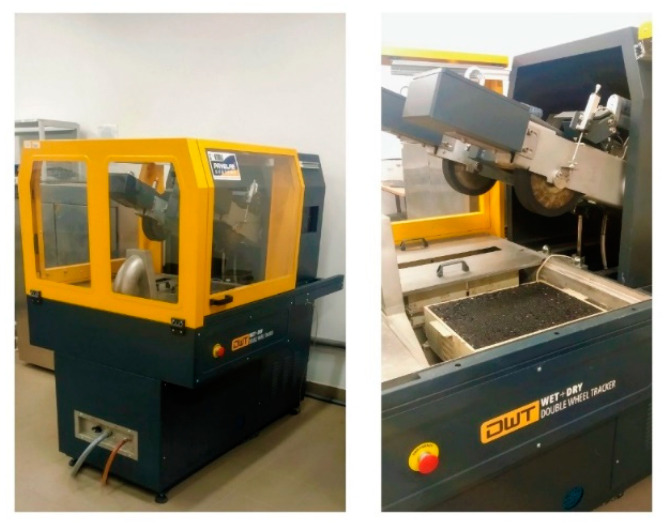
DWT test device and sample setup.

DWT test device with the mounted sample is shown in Figure 2.

## 4. Results and Discussion

### 4.1. IT-CY Stiffness Modulus

The results of the stiffness modulus test with the descriptive statistics are presented in Table 3.

The obtained results of stiffness modulus are similar to the test results described in publications [40,41]. Mixtures with binder modified with SBS and CR achieved higher modulus values compared to mixtures with unmodified binder. This proves that they are less susceptible to traffic loads and show higher resistance to deformation. The addition of RG causes a decrease in the stiffness modulus of the mixtures in direct proportion to the amount of granulate introduced. This proves that replacing the aggregate with RG makes the mixture more flexible. Therefore, it can be predicted that they will more effectively dampen vehicle vibrations and road noise.

Based on the results of the tests presented in Table 3, it was found that the highest values of the modulus at 5 °C were obtained for the mixtures: SMA8 with CRM-10 and SBSM-2+CRM-10 (10,183 MPa and 10,156 MPa, respectively) and SMA8 LA with bitumen 50/70 and SBSM-2+CRM-10 (7894 MPa and 7526 MPa, respectively). The lowest values of the modulus at 35 °C were obtained for SMA8 LA (30% RG) with bitumen 50/70 and CRM-10 (28 MPa and 27 MPa, respectively). It was found that the highest dispersion of test results (CoV) was obtained at the temperatures of 25 °C and 35 °C. The SMA8 LA mixtures with the addition of 10%, 20% and 30% rubber granulate with bitumen 50/70 showed scatter of results CoV = 17.6–20.2, SMA8 LA (20% RG) with SBSM-5 (35 °C) CoV = 20.6, SMA8 LA with the addition of 20% RG, and 30% RG with SBSM-2+CRM-10—CoV = 16.6–20.8.

**Figure 3:**
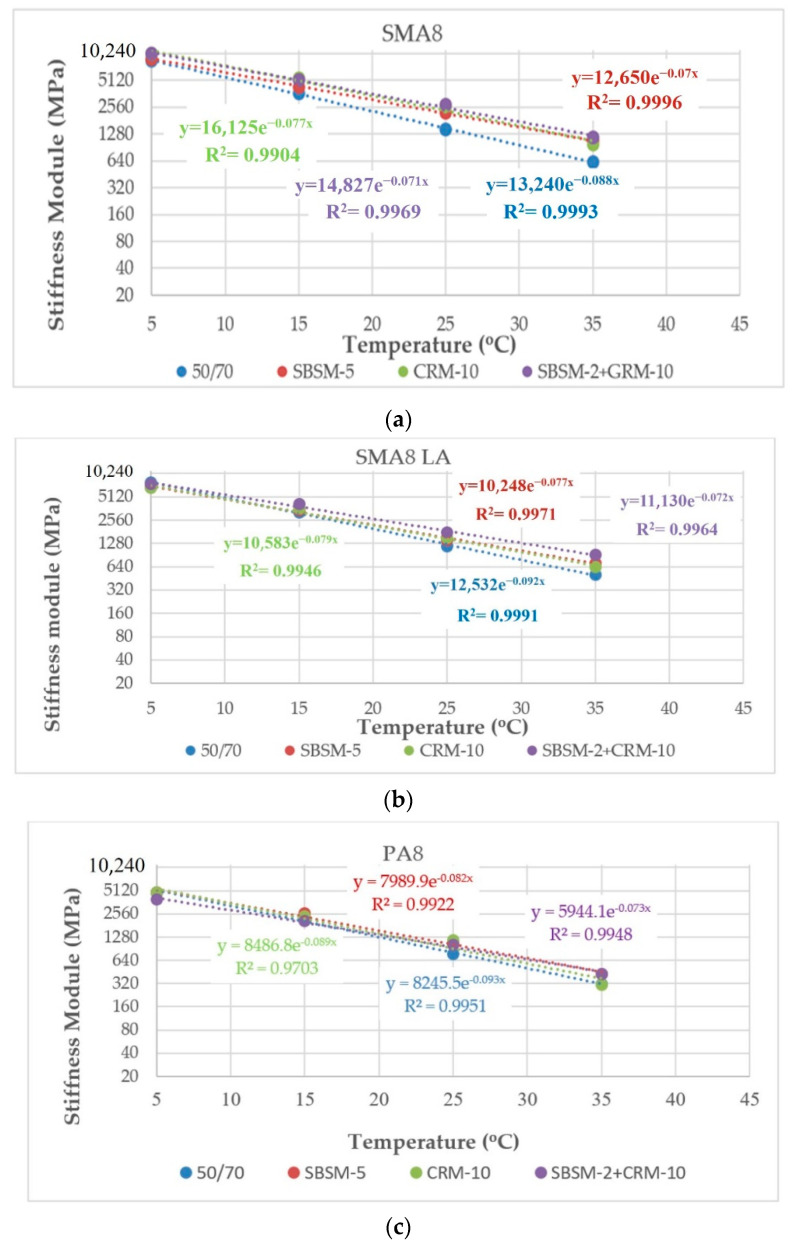

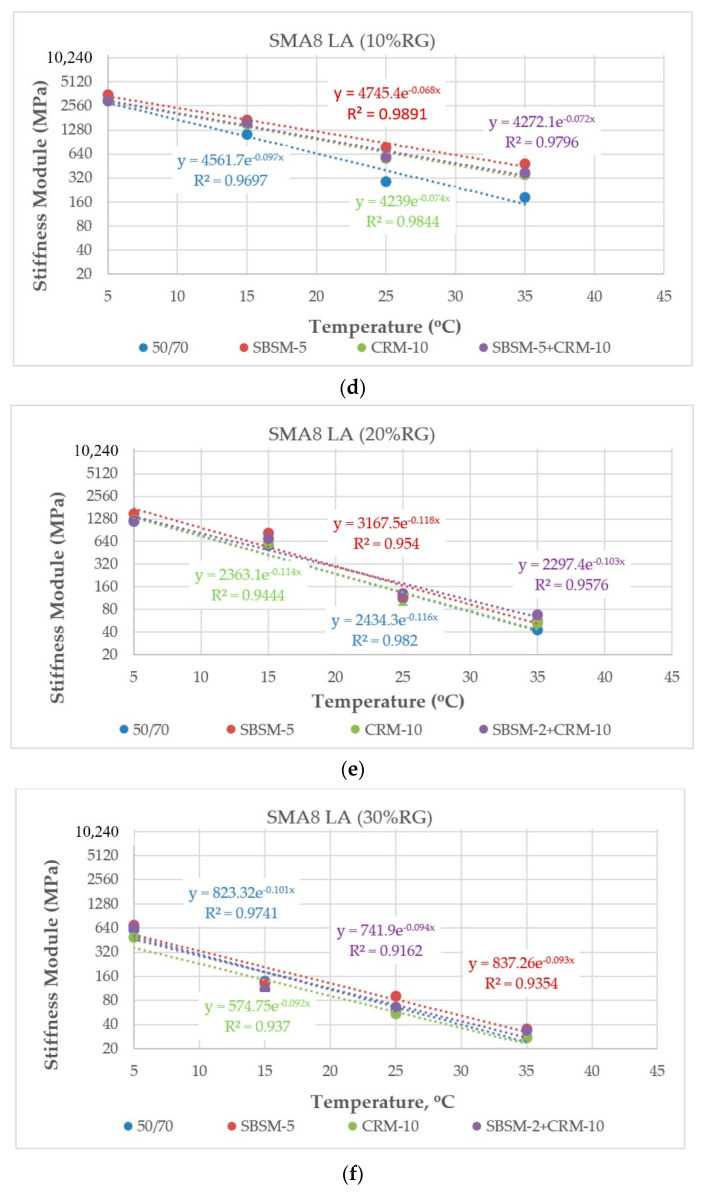
IT-CY stiffness modulus as a function of temperature for mixtures: (**a**) SMA8; (**b**) SMA8 LA; (**c**) PA8; (**d**) SMA8 LA(10%RG); (**e**) SMA8 LA(20%RG); and (**f**) SMA8 LA(30%RG).

Figure 3 shows the results of IT-CY stiffness modulus as a function of temperature.

The results presented in Figure 3 show that the type of modifier and the amount of RG affect the stiffness modulus of the tested mixtures. SMA8 and SMA8 LA with modified bitumen SBSM-5 showed the lowest temperature sensitivity. PA8 and SMA8 LA (20% RG) mixtures are characterized by the lowest temperature sensitivity when using the SBSM-2+CRM-10 binder. The lowest temperature sensitivity among the mixtures with the addition of RG has SMA8 LA (10% RG) and SMA8 LA (30% RG) with rubber-asphalt binder CRM-10. All analyzed mixtures with the reference bitumen 50/70 showed higher temperature sensitivity compared to the bitumen modified with SBS and CR. The results of the research presented in [5,41] also prove that mixtures with binders modified with SBS and CR are characterized by lower or comparable sensitivity to changes in stiffness modulus as a function of temperature compared to mixtures with unmodified bitumen.

The analysis of the influence of the addition of 10%, 20% and 30% RG on the change of the IT-CY stiffness modulus of asphalt mixtures in relation to the reference mixture SMA8 LA with bitumen 50/70 is presented in Table 4.

Table 4 shows that the greatest changes in the stiffness modulus values in relation to the reference mixture were observed when using 20% and 30% RG. A significant drop in stiffness (about 90%) was obtained in these mixtures, which means that the influence of the type of bitumen was reduced, and the amount of RG added determined the value of the test result. The greatest changes in IT-CY values were observed for the SMA8 LA (30% RG) mixture with GRM-10 binder (decrease by 95%) and bitumen 50/70 (decrease by 94%).

The second-degree polynomial was used to describe the changes in the stiffness modulus:

(5) $$\;\;\;\;Z=a_{0}+a_{1}X_{1}+a_{2}X_{2}+a_{3}X_{3}+a_{4}X_{1}X_{2}+a_{5}X_{1}X_{3}+a_{6}X_{2}X_{3}+a_{7}X_{1}^{2}+a_{8}X_{2}^{2}+a_{9}X_{3}^{2}$$

where

*Z*—analyzed parameter of the mixture (IT-CY stiffness modulus);
$a_{0}-a_{9}$—regression coefficients;
*X*_1_—type of mixture;
*X*_2_—type of binder;
*X*_3_—temperature (°C).

The statistical analysis of the obtained results according to ANOVA started with the factorial significance test (Statistica Software, version 13, TIBCO Software Inc., Palo Alto, CA, USA). The results of this analysis are presented in Table 5.

It can be clearly stated based on the analysis of the parameters that the temperature, mixture type and modifier type are important factors affecting the stiffness modulus, because the *p*-value is lower than the assumed significance level α = 0.05 (*p*-value <0.05) (Table 5).

Analyzing the quadratic term of the modifier type (Type of Binder (Q)) and the factor describing the interaction of the modifier type and temperature (2L*3L), no significant influence was found on the values of stiffness modulus (*p*-value greater than α = 0.05).

The values describing the parameters of the regression model are summarized in Table 6.

Based on the analysis, it was observed that the value of the corrected determination coefficient was $R_{adj}^{2}$ = 97%, which proves that the model was correctly adopted (Table 6).

The developed model of the IT-CY stiffness modulus can be presented using the dependence (6).

(6) $$ITCY=4006.42-2221.01\;TM+89.25\;TM^{2}+2117.72\;TB-41.91\;TB^{2}-777.06\;Temp\;+4.37\;Temp^{2}-68.75\;TM\;TB+53.46\;TM\;Temp+0.95\;TB\;Temp,$$

where

*TM*—type of mixture: SMA8 = 6, SMA8 LA = 7, PA8 = 8, SMA8 LA (10%RG) = 9, SMA8 LA (20%RG) = 10, SMA8 LA (30%RG) = 11;
*TB*—type of binder: 50/70 = 12, SBSM-5 = 13, CRM-10 = 14, SBSM-2+CRM-10 = 15;
*Temp*—test temperature.

**Figure 4:**
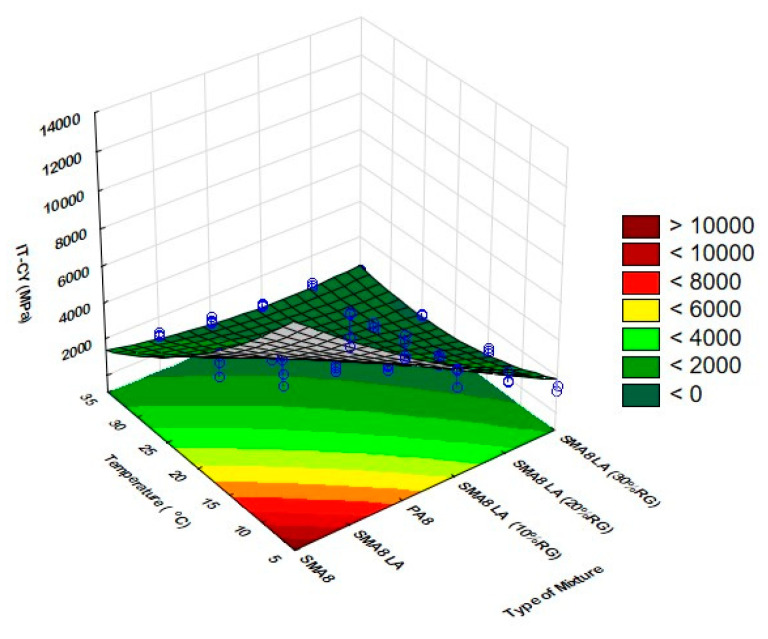
Stiffness modulus as a function of temperature and mixture type.

The selected graphical interpretation of the IT-CY stiffness modulus change as a function of temperature and mixture type is presented in Figure 4.

### 4.2. Rutting Resistance by British and Belgian Method

**Figure 5:**
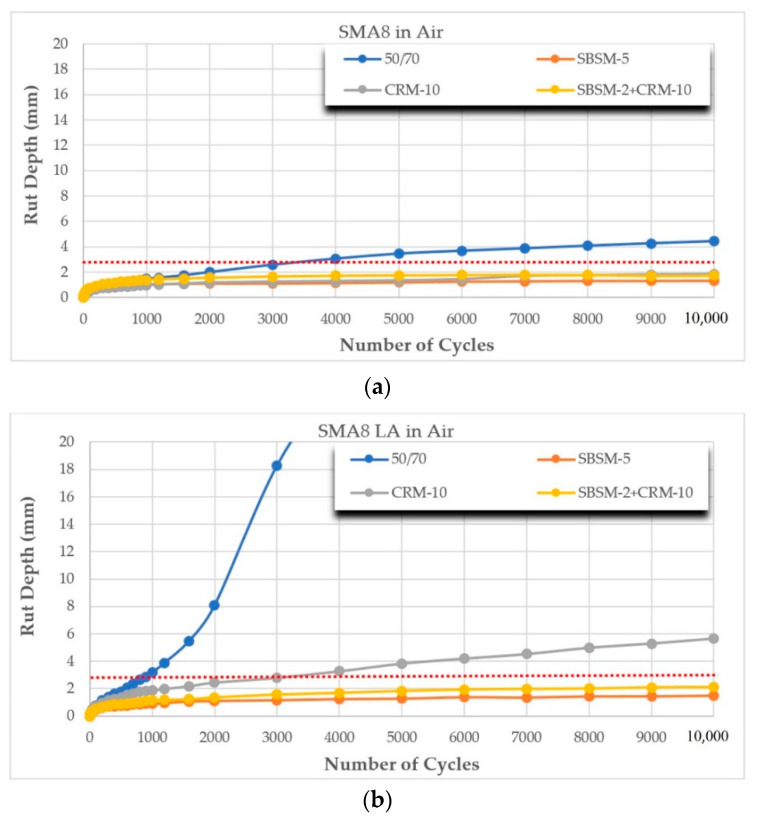

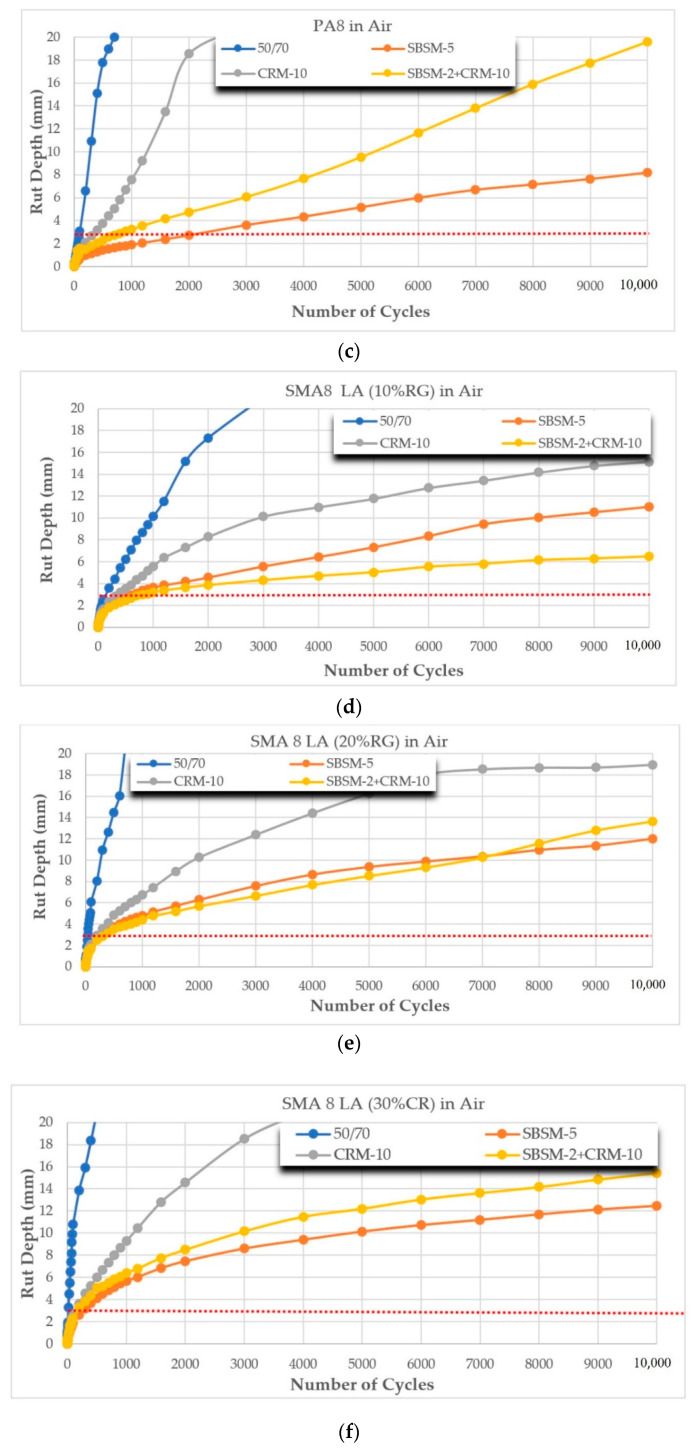
Dependence of rut depth on the number of cycles and mixture type using the British method: (**a**) SMA8; (**b**) SMA8 LA; (**c**) PA8; (**d**) SMA8 LA (10%RG); (**e**) SMA8 LA (20%RG); and (**f**) SMA8 LA (30%RG).

**Figure 6:**
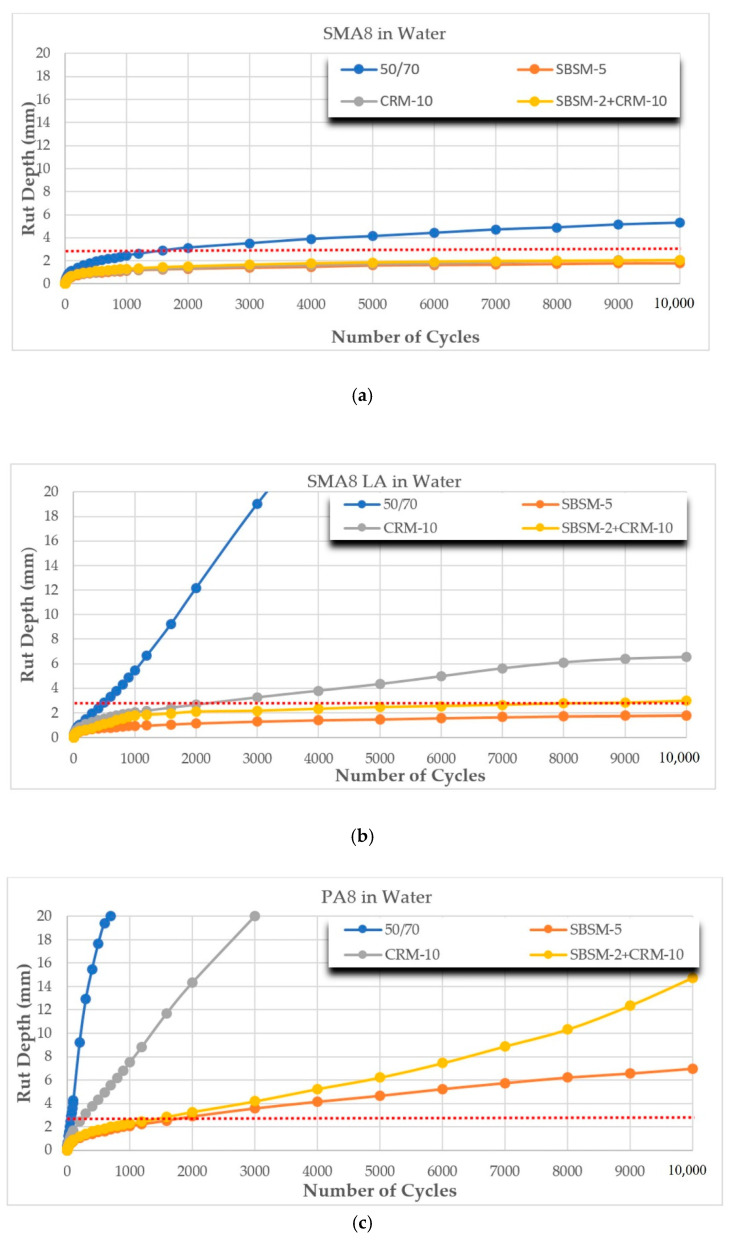

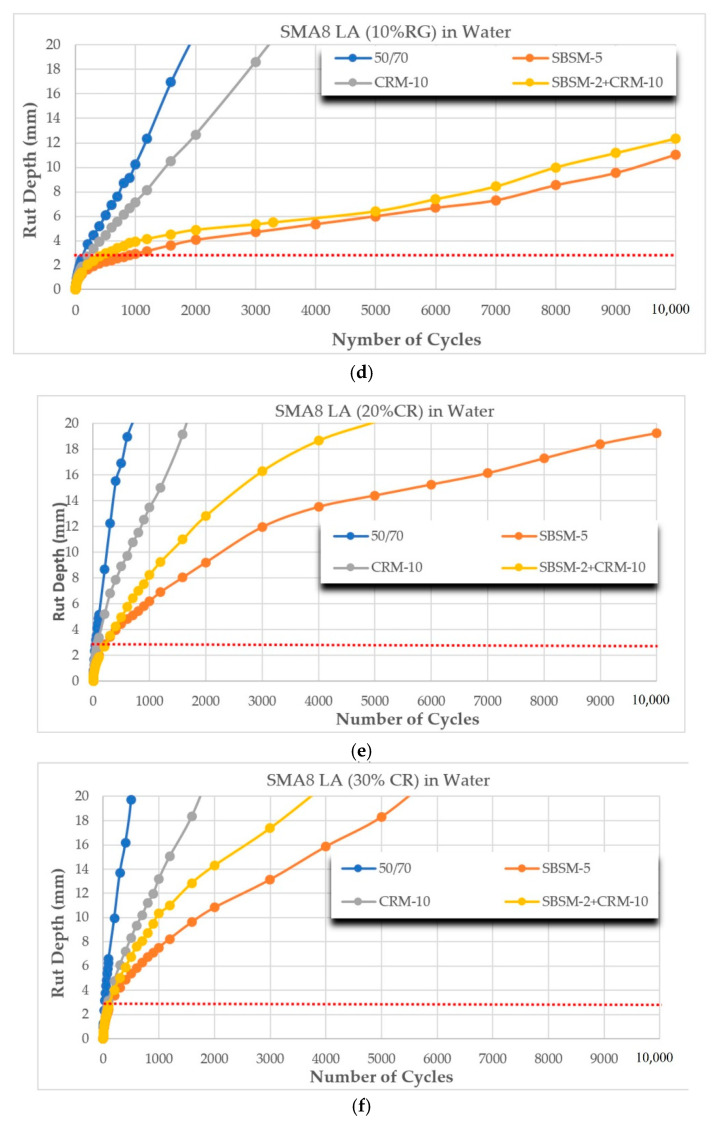
Dependence of rut depth on the number of cycles and mixture type using the Belgian method: (**a**) SMA8; (**b**) SMA8 LA; (**c**) PA8; (**d**) SMA8 LA (10%RG); (**e**) SMA8 LA (20%RG); and (**f**) SMA8 LA (30%RG).

The results of rutting resistance tests of the analyzed mixtures using the British and Belgian methods are presented in Figure 5a–f and Figure 6a–f, and in Table 7. The red line marks the maximum allowable rut depth (2.8 mm) after 10,000 cycles according to [42]. The table below the graphs presents the calculated values of the WTS and PRD rutting parameters. The requirements [42] permit the following limits: WTS_air_ ≤ 0.15 and PRD_air_ ≤ 7.0.

The rutting phenomenon occurs mainly at high temperatures. Therefore, it is important to use binders with appropriate viscoelastic properties at temperatures above 60 °C. On the basis of the rutting results presented in Figure 5a–f and Figure 6a–f and in Table 7, it was found that the test type, mixture type and modifier type have a significant influence on the rutting resistance of asphalt mixture. It was established that the test in water (Belgian method) gives higher values of WTS_w_ and PRD_w_ indexes compared to the results of tests in the air (British method). The highest (unfavorable) results of the WTS_w_ index were obtained for SMA8 LA (10% RG), SMA8 LA (20% RG) and SMA8 LA (30% RG) mixtures with SBSM-2+CRM-10 binder, and the WTS_w_ index increased by 334%, 215% and 89% compared to WTS_air_ values, respectively. The highest (unfavorable) values of the percentage of rut depth PRD_w_ were obtained for SMA8 LA (20% RG) and SMA8 LA (30% RG) mixtures with SBSM-5 bitumen, and PRD_w_ increased by 60% and 65% compared to PRD_air_ values, respectively.

Different results of the WTS and PRD tests were obtained for the PA8 mixture. With these mixtures, rutting indexes were lower (favorable) when tested in water. This can be explained by the fact that water that penetrated into the open pores (air void content in PA8 was 24%) acted like “shock absorber”. Thus, it improved the resistance of PA8 mixtures to rutting. For PA8 mixtures with CRM-10 binder, the WTS_w_ index decreased by 34% compared to WTS_air_.

Apart from the test method, the type of binder has a significant influence on the rutting resistance. The best results were obtained for mixtures with SBSM-5 modified bitumen compared to the reference bitumen 50/70. The greatest change in parameters was recorded for the SMA8 LA mixtures, where the WTS_w_ index decreased from 6.97 to 0.06, and the PRD_air_ index decreased from 50.00 to 3.93. In the case of PA8 mixtures, WTS_air_ decreased from 37.79 to 0.61, and PRD_w_ from 50.00 to 17.26.

The rutting resistance tests showed that the amount of addition RG had a direct proportionate effect on the WTS and PRD parameters. The greater the replacement level of the mineral aggregate with the RG, the greater the elasticity of the mixture, and the more significant the deterioration of the analyzed parameters. Taking as an example SMA8 LA mixture with SBSM-5 binder for which WTS_air_ equals 0.05 and PRD_air_ equals 3.93, after adding 30% GR (SMA8 LA (30% RG)), the rutting parameters increased (unfavorable) to the following values: WTS_air_—2.11 and PRD_air_—30.31.

A significant improvement in the rut resistance of asphalt-rubber mixtures com-pared to mixtures with conventional binders was also achieved in [43,44]. The authors showed that the addition of CR to bitumen over 10% increases binder stiffness and viscosity. It leads to an increase in stiffness and the rate of increase in the rut depth. The addition of SBS copolymer, as reported by the authors of the publications [34,43,45], in-creases the rut resistance of the mixtures at least twice. When SBS is added to the standard binder, a load-bearing butadiene network is formed that increases its viscosity, stiffness and elasticity.

The second-degree polynomial was used to describe changes in rutting parameters:

(7) $$Z=a_{0}+a_{1}X_{1}+a_{2}X_{2}+a_{3}X_{1}X_{2}+a_{4}X_{1}^{2}+a_{5}X_{2}^{2}$$

where

*Z*—the analyzed parameter (WTS_air_, PRD_air_, WTS_w_ i PRD_w_);

$a_{0}-a_{5}$—regression coefficients;

*X*_1_—type of mixture;

*X*_2_—type of binder.

The results of the statistical analysis of the influence of mixture type and modifier type on the rutting indexes are presented in Table 8, while Table 9 presents the estimation of the parameters of the model describing the relationship between the type of binder and mixture and the rutting indexes.

The statistical analysis proves that the WTS_air_ value is influenced by the modifier type, the PRD_air_ and PRD_w_ values are strongly affected by the mixture type and the WTS_w_ value is influenced by both the mixture type and the modifier type. This is evidenced by the *p*-value (Table 8), the value of which is lower than the assumed significance level α = 0.05 (*p*-value < 0.05). However, it should be noted that there is no interaction between the influence of the mixture type and modifier type (1L * 2L) on the WTS and PRD rutting resistance parameters (*p*-value greater than α = 0.05). Low values of the determination coefficient result from the high variability of the results in the analyzed groups.

The developed model of the analyzed characteristics can be expressed using the Equations (8)–(11).

(8) $$\;\;\;\;\;\;\;\;\;\;\;\;\;\;\;\;\;\;\;\;\;\;\;\;\;\;\;\;WTS_{air}=900.203+29.945\;TM-0.313\;TM^{2}-113.386\;TB+3.427\;TB^{2}-1.3010\;TM\;TB$$

(9) $$\;\;\;\;\;\;\;\;\;\;\;\;\;\;\;\;\;\;\;\;\;\;\;\;\;\;\;PRD_{air}=1144.160+33.324\;TM-2.096\;TM^{2}-142.485\;TB+3.826\;TB^{2}-0.520\;TM\;TB$$

(10) $$\;\;\;\;\;\;\;\;\;\;\;\;\;\;\;\;\;\;\;WTS_{w}=747.822+22.099\;TM+0.055\;TM^{2}-93.244\;TB+2.841\;TB^{2}-1.178\;TM\;TB$$

(11) $$\;\;\;\;\;\;\;\;\;\;\;\;\;\;\;\;\;\;\;\;\;PRD_{w}=980.219+14.923\;TM-1.730\;TM^{2}-116.431\;TB+2.917\;TB^{2}+1.328\;TM\;TB$$

where

*TM*—type of mixture: SMA8 = 6, SMA8 LA =7, PA8 = 8, SMA8 LA (10%RG) = 9, SMA8 LA (20%RG) = 10, SMA8 LA (30%RG) = 11;
*TB*—type of binder: 50/70 = 12, SBSM-5 = 13, CRM-10 = 14, SBSM-2+CRM-10 = 15.

**Figure 7:**
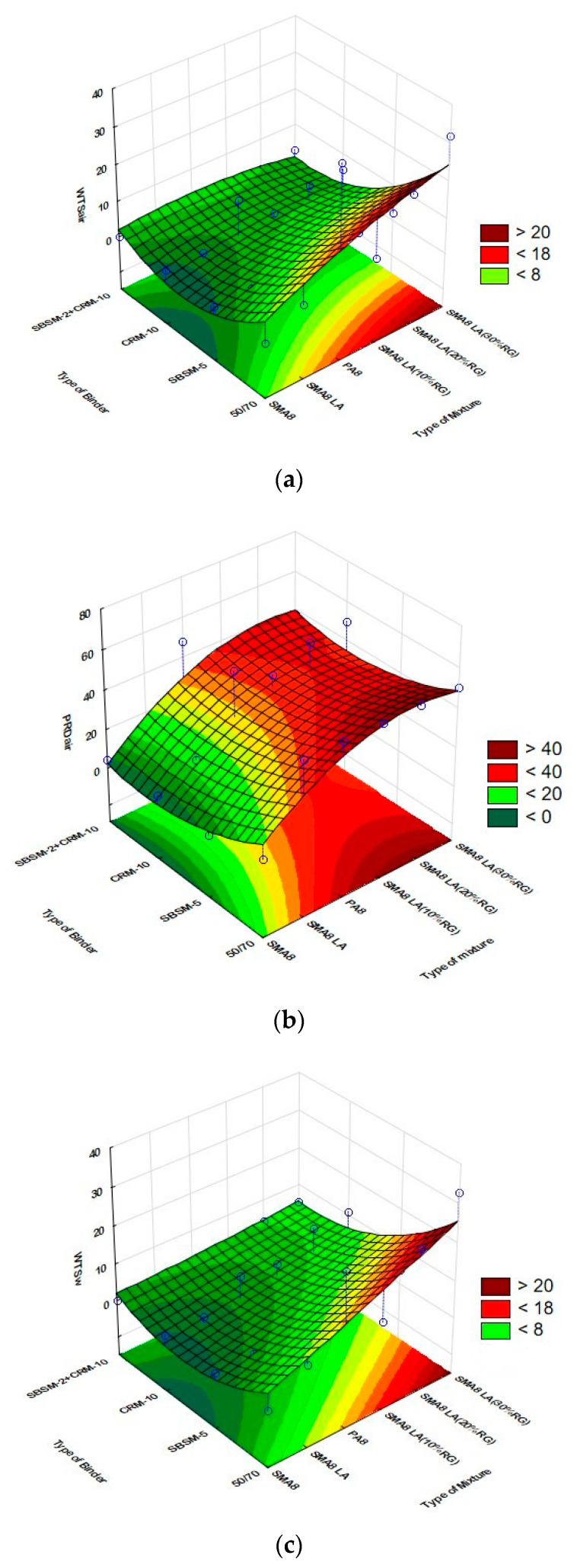

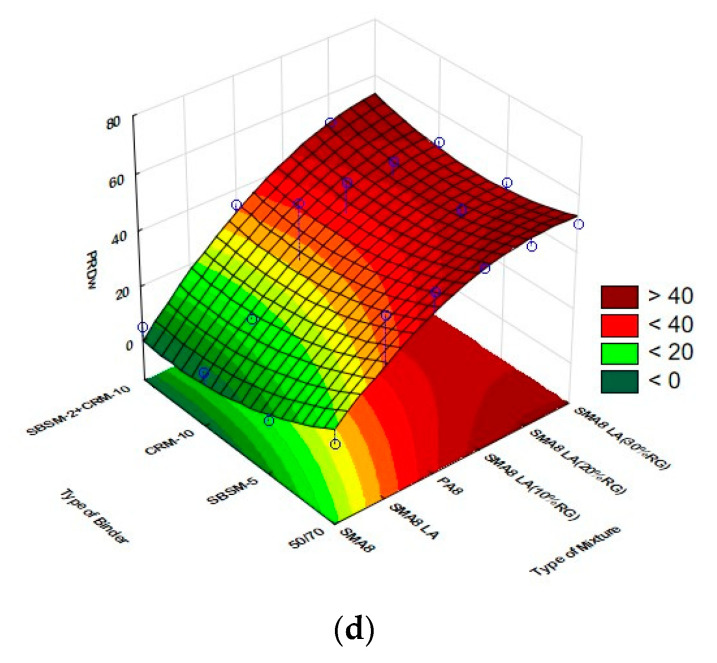
Graphical interpretation of the adopted models: (**a**) WTS_air_, (**b**) PRD_air_, (**c**) WTS_w_ and (**d**) PRD_w,_ as a function of mixture type and modifier type.

Graphical interpretation of the adopted WTS and PRD models for different types of analyzed mixtures and bituminous binders is shown in Figure 7.

## 5. Conclusions

Based on the tests of the stiffness modulus and rutting resistance of grit mastic mixtures SMA8, SMA8 LA and porous asphalt PA8, with binders modified with SBS copolymer or crumb rubber, or combined modification with SBS and crumb rubber, the following conclusions were formulated:

1. The type of modifier used has a significant effect on the stiffness of asphalt mixtures and their temperature sensitivity confirmed by the change in the value of the stiffness modulus as a function of temperature. The highest increase was obtained at the temperature of 35 °C for SMA8 LA mixtures with 10% rubber granulate: by 163% for the binder modified with SBS copolymer, by 92% for the binder modified with crumb rubber and by 104% for the modification with 2% SBS + 10% crumb rubber, compared to mixtures with bitumen 50/70.
2. Replacing coarse aggregate in the mixture with rubber granulate in an amount exceeding 20% (by volume) causes a significant decrease in the stiffness modulus. The greatest changes were observed in the case of SMA8 LA (30% rubber granulate) with a binder modified with 10% crumb rubber. The decrease in IT-CY stiffness modulus in this case was 95% compared to the reference mixture with bitumen 50/70.
3. In SMA mixtures, their rutting resistance was found to be lower in Belgian test (in water) compared to the British test (in air). The SMA8 LA (10% rubber granulate) mixture showed an increase in the WTS_w_ rut depth by 334% in relation to the results measured in the air. Percentage of rut depth PRD_w_ of SMA8 LA (30% rubber granulate) mixture increased by 65% compared to PRD_air_.
4. The opposite effect was achieved for porous asphalt. Water present in the open pores of PA mixtures during the test is likely to act as a “shock absorber”, partially taking the load while improving the rutting resistance of these mixtures. The WTS_w_ index decreased by 34% compared to WTS_air_ for samples with binder containing 10% crumb rubber. Percentage of rut depth decreased by 27% when binder was modified with a combined SBS copolymer and crumb rubber was applied.
5. The addition of rubber granulate directly affects the WTS and PRD parameters. The higher the replacement ratio of mineral aggregate with rubber granulate, the more the rutting parameters deteriorate. For example, for SMA8 LA mixture with a 5% copolymer modified binder, WTS_air_ is 0.05 and PRD_air_ is 3.93. If the addition of rubber granulate in SMA LA is 30%, the rutting indexes are: WTS_air_ = 2.11 and PRD_air_ = 30.31.

## Figures and Tables

**Table 1:** Technical properties of modified binders.

Indexes	Units of Measurement	Type of Binder							
		50/70		SBSM-5		CRM-10		SBSM-2+CRM-10	
		(a)	(b)	(a)	(b)	(a)	(b)	(a)	(b)
Penetration	0.1 mm								
5 °C		11.4	8.4	8.7	6.1	8.7	6.8	7.7	5.6
15 °C		32.8	20.3	20.6	16.2	19.5	14.1	16.2	13.1
25 °C		58.3	44.3	40.2	30.1	40.0	27.8	30.6	24.8
Softening Point	°C	50.8	56.3	78.6	77.8	60.6	68.2	70.7	77.8
Fraass Breaking Point	°C	−14.7	−12.9	−19.3	−17.3	−16.1	−15.5	−17.9	−16.5
Dynamic Viscosity	Pa s								
90 °C		11.3	19.3	224.4	258.7	83.6	265.4	292.2	574.1
110 °C		2.2	3.6	25.1	27.7	13.4	39.3	43.7	74.9
135 °C		0.5	0.7	2.5	3.6	2.1	4.8	5.4	8.8

where: (a)—before RTFOT; (b)—after RTFOT.

**Table 2:** Characteristics of the tested mixtures.

Properties	Type of Mixture					
	PA8	SMA8	SMA8 LA	SMA8 LA (10% RG)	SMA8 LA (20% RG)	SMA8 LA (30% RG)
Air void content (%)	23.8	2.89	10.56	11.64	11.99	15.0
Binder content (%)	6.3	6.8	6.8	8.0	10.0	12.0
Bulk density (Mg/m^3^)	1.954	2.378	2.261	2.045	1.819	1.651

**Table 3:** The results of the IT-CY stiffness modulus test in temperatures: 5 °C, 15 °C, 25 °C and 35 °C.

Type of Mixture	Type of Binder	Value of Statistical Parameters of Stiffness Modules			
		Temperature	Mean	Standard Deviation	Coefficient of Variation (%)
SMA8	50/70	5	8447	467.3	5.5
		15	3679	249.0	6.7
		25	1431	97.7	6.8
		35	624	11.6	1.8
	SBSM-5	5	8945	356.5	3.9
		15	4311	300.5	6.9
		25	2216	139.5	6.2
		35	1065	134.6	12.64
	CRM-10	5	10,183	720.7	7.0
		15	5314	214.4	4.0
		25	2602	58.3	2.2
		35	976	51.1	5.2
	SBSM-2+CRM-10	5	10,156	707.7	6.9
		15	5096	133.5	2.6
		25	2693	122.9	4.5
		35	1174	152.8	13.0
SMA8 LA	50/70	5	7894	205.3	2.6
		15	3207	224.1	6.9
		25	1181	147.9	12.5
		35	505	24.5	4.8
	SBSM-5	5	6808	377.3	5.5
		15	3464	237.1	6.8
		25	1420	90.1	6.3
		35	710	21.6	3.0
	CRM-10	5	6633	424.1	6.3
		15	3557	146.2	4.1
		25	1490	33.7	2.2
		35	633	20.3	3.2
	SBSM-2+CRM-10	5	7526	345.9	4.5
		15	4082	317.4	7.7
		25	1754	121.1	6.9
		35	910	19.2	2.1
PA8	50/70	5	4840	322.1	6.6
		15	2303	120.1	5.2
		25	770	34.7	4.5
		35	313	12.5	4.0
	SBSM-5	5	4860	355.5	7.3
		15	2575	163.5	6.3
		25	1092	44.0	4.0
		35	419	18.5	4.4
	CRM-10	5	4794	173.9	3.6
		15	2350	83.8	3.5
		25	1176	146.9	12.4
		35	307	18.0	5.8
	SBSM-2+CRM-10	5	3901	287.1	7.3
		15	2067	166.1	8.1
		25	1018	55.9	5.4
		35	427	19.0	4.4
SMA8 LA (10% RG)	50/70	5	2996	150.2	5.0
		15	1126	91.1	8.0
		25	291	58.5	20.1
		35	184	22.0	12.0
	SBSM-5	5	3537	153.2	4.3
		15	1712	56.4	3.2
		25	769	51.7	6.7
		35	483	27.6	5.6
	CRM-10	5	2962	161.0	5.4
		15	1508	94.2	6.2
		25	566	70.2	12.4
		35	353	26.0	7.3
	SBSM-2+CRM-10	5	2983	195.6	6.5
		15	1588	41.4	2.6
		25	581	47.4	8.1
		35	376	25.6	6.8
SMA8 LA (20% RG)	50/70	5	1196	71.6	5.9
		15	563	65.1	11.5
		25	112	113.0	4.8
		35	43	7.4	17.6
	SBSM-5	5	1484	132.7	5.4
		15	830	56.1	6.7
		25	119	7.7	6.4
		35	56	11.5	20.6
	CRM-10	5	1235	79.8	6.4
		15	602	31.3	5.2
		25	85	10.0	11.8
		35	52	6.3	12.1
	SBSM-2+CRM-10	5	1202	42.0	3.4
		15	699	36.9	5.2
		25	132	21.9	16.6
		35	69	12.1	17.7
SMA8 LA (30% RG)	50/70	5	609	97.0	15.9
		15	140	15.8	11.2
		25	60	12.1	20.2
		35	28	5.3	19.1
	SBSM-5	5	691	85.1	12.3
		15	132	21.6	16.4
		25	90	3.3	3.7
		35	35	5.2	14.9
	CRM-10	5	485	58.4	12.0
		15	96	13.9	14.4
		25	54	4.3	7.9
		35	27	2.9	10.8
	SBSM-2+CRM-10	5	655	53.4	8.1
		15	111	16.9	15.3
		25	66	6.2	9.4
		35	34	7.0	20.8

**Table 4:** Change of IT-CY stiffness modulus in relation to amount of RG in temperature 35 °C.

Type of Mixture	Type of Binder	Stiffness Modules Change (MPa)
SMA8 LA	50/70	505 (0%)—reference
	SBSM-5	710 (+41%)
	CRM-10	633 (+25%)
	SBSM-2+CRM-10	910 (+80%)
SMA8 LA (10%RG)	50/70	184 (−64%)
	SBSM-5	483 (−4%)
	CRM-10	353 (−30%)
	SBSM-2+CRM-10	376 (−26%)
SMA8 LA (20%RG)	50/70	43 (−91%)
	SBSM-5	56 (−89%)
	CRM-10	52 (−90%)
	SBSM-2+CRM-10	69 (−86%)
SMA8 LA (30%RG)	50/70	28 (−94%)
	SBSM-5	35 (−93%)
	CRM-10	27 (−95%)
	SBSM-2+CRM-10	34 (−93%)

**Table 5:** Assessment of the significance of the influence of temperature, mixture type and modifier type on changes in the stiffness modulus using ANOVA test.

Effect	Variable: ITCY (MPa); R^2^ = 0.9692; $R_{\mathrm{adj}}^{2}$ = 0.96598; Error MS = 195,455			
	SS	MS	F	*P*
(1) Type of Mixture (L)	196,457,349	196,457,349	1005.128	0.000000
Type of Mixture (Q)	4,757,592	4,757,592	24.341	0.000004
(2) Type of Binder (L)	874,866	874,866	4.476	0.037265
Type of Binder (Q)	168,650	168,650	0.863	0.355539
(3) Temperature (L)	206,716,650	206,716,650	1057.618	0.000000
Temperature (Q)	18,324,926	18,324,926	93.755	0.000000
1L*2L	1,654,081	1,654,081	8.463	0.004613
1L*3L	100,039,110	100,039,110	511.827	0.000000
2L*3L	13,525	13,525	0.069	0.793137
Error	16,809,128	195,455		
Total SS	545,815,879			

where: Q—quadratic; L—linear.

**Table 6:** Parameters of dependence model of the stiffness modulus on temperature, mixture type and modifier type.

Effect	Variable: ITCY (MPa); R2 = 0.9692; $R_{\mathrm{adj}}^{2}$ = 0.96598; Error MS = 195,455					
	Regression Coefficients	Std. Error	t	*p*-Value	−95% Conf. Lmt	+95% Conf. Lmt
Intercept	4006.42	14,340.78	0.2794	0.780630	−24,502.1	32,514.95
(1) Type of Mixture (L)	−2221.01	518.19	−4.2861	0.000047	−3251.1	−1190.89
Type of Mixture (Q)	89.25	18.09	4.9337	0.000004	53.3	125.21
(2) Type of Binder (L)	2117.72	1594.14	1.3284	0.187547	−1051.3	5286.76
Type of Binder (Q)	−41.91	45.12	−0.9289	0.355539	−131.6	47.79
(3) Temperature (L)	−777.06	68.82	−11.2914	0.000000	−913.9	−640.25
Temperature (Q)	4.37	0.45	9.6827	0.000000	3.5	5.27
1L*2L	−68.75	23.63	−2.9091	0.004613	−115.7	−21.77
1L*3L	53.46	2.36	22.6236	0.000000	48.8	58.16
2L*3L	0.95	3.61	0.2631	0.793137	−6.2	8.13

where: Q—quadratic; L—linear.

**Table 7:** The results of rutting resistance tests of the analyzed mixtures using the British and Belgian methods.

Type of Mixture	Type of Binder			
	50/70	SBSM-5	CRM-10	SBSM-2+CRM-10
**WTS/PRD in air**				
SMA8	0.20/11.02	0.03/3.47	0.06/4.49	0.01/4.37
SMA8 LA	5.48/50.00	0.05/3.93	0.29/13.57	0.06/5.63
PA8	37.79/50.00	0.61/19.55	10.23/50.00	1.89/47.87
SMA8 LA (10%RG)	8.17/50.00	0.55/24.37	2.24/39.28	0.31/16.49
SMA8 LA (20%RG)	20.78/50.00	1.32/29.99	5.38/47.04	1.27/34.93
SMA8 LA (30%RG)	31.88/50.00	2.11/30.31	5.39/50.00	2.65/37.52
**WTS/PRD in water**				
SMA8	0.23/13.94	0.04/4.46	0.08/5.22	0.06/5.12
SMA8 LA	6.97/50.00	0.06/4.45	0.40/16.25	0.08/7.53
PA8	26.36/50.00	0.50/17.26	6.74/50.00	1.66/34.74
SMA8 LA (10%RG)	8.37/50.00	1.08/28.81	5.43/50.00	1.35/32.99
SMA8 LA (20%RG)	23.05/50.00	1.85/47.86	10.76/50.00	4.00/50.00
SMA8 LA (30%RG)	32.82/50.00	3.91/50.00	10.94/50.00	5.02/50.00

**Table 8:** Assessment of the influence of mixture type and modifier type on WTS_air_, PRD_air_ and WTS_w_ i PRD_w_.

**Effect**	**Variable: WTS_air_; R^2^ = 0.54327; ** $R_{\mathrm{adj}}^{2}$ **= 0.4164; Error MS = 59.81792**			
	**SS**	**MS**	**F**	***P***
(1) Type of Mixture (L)	202.295	202.2951	3.38185	0.082475
Type of Mixture (Q)	14.643	14.6431	0.24480	0.626749
(2) Type of Binder (L)	631.904	631.9037	10.56379	0.004443
Type of Binder (Q)	281.791	281.7909	4.71081	0.043597
1L*2L	150.114	150.1137	2.50951	0.130572
Error	1076.723	59.8179		
Total SS	2357.469			
**Effect**	**Variable: PRD_air_; R^2^ = 0.61231; $R_{\mathrm{adj}}^{2}$ = 0.50462; Error MS = 168.3381**			
	**SS**	**MS**	**F**	***P***
(1) Type of Mixture (L)	3234.400	3234.400	19.21371	0.000358
Type of Mixture (Q)	655.989	655.989	3.89685	0.063926
(2) Type of Binder (L)	520.331	520.331	3.09098	0.095719
Type of Binder (Q)	351.261	351.261	2.08664	0.165775
1L*2L	23.677	23.677	0.14065	0.712014
Error	3030.086	168.338		
Total SS	7815.744			
**Effect**	**Variable: WTS_w_; R^2^ = 0.63418; ** $R_{\mathrm{adj}}^{2}$ **= 0.53256; Error MS = 37.379**			
	**SS**	**MS**	**F**	***P***
(1) Type of Mixture (L)	410.188	410.1882	10.97376	0.003872
Type of Mixture (Q)	0.450	0.4504	0.01205	0.913810
(2) Type of Binder (L)	440.681	440.6810	11.78953	0.002963
Type of Binder (Q)	193.663	193.6630	5.18106	0.035285
1L*2L	121.387	121.3871	3.24747	0.088306
Error	672.822	37.3790		
Total SS	1839.192			
**Effect**	**Variable: PRD_w_; R^2^ = 0.76691 $R_{\mathrm{adj}}^{2}$ = 0.70217; Error MS = 108.761**			
	**SS**	**MS**	**F**	***P***
(1) Type of Mixture (L)	5359.360	5359.360	49.27651	0.000001
Type of Mixture (Q)	447.183	447.183	4.11161	0.057657
(2) Type of Binder (L)	276.121	276.121	2.53878	0.128489
Type of Binder (Q)	204.261	204.261	1.87807	0.187404
1L*2L	154.382	154.382	1.41946	0.248965
Error	1957.697	108.761		
Total SS	8399.004			

where: Q—quadratic; L—linear.

**Table 9:** Parameters of the model of relationship between the type of binder and mixture and rutting indexes.

**Effect**	**Variable: WTS_air_; R^2^ = 0.54327; ** $R_{\mathrm{adj}}^{2}$ **= 0.4164; Pure Error MS = 59.81792**					
	**Regression Coefficients**	**Std. Error**	**T**	***p*** **-value**	**−95% Conf.** **Lmt**	**+95% Conf.** **Lmt**
Intercept	900.203	499.5835	1.80191	0.088333	−149.383	1949.789
(1) Type of Mixture (L)	29.945	18.0549	1.65854	0.114531	−7.987	67.877
Type of Mixture (Q)	−0.313	0.6329	−0.49477	0.626749	−1.643	1.017
(2) Type of Binder (L)	−113.386	55.7189	−2.03496	0.056855	−230.447	3.675
Type of Binder (Q)	3.427	1.5787	2.17044	0.043597	0.110	6.743
1L*2L	−1.310	0.8268	−1.58414	0.130572	−3.047	0.427
**Effect**	**Variable: PRD_air_; R^2^ = 0.61231; ** $R_{\mathrm{adj}}^{2}$ **= 0.50462; Pure Error MS = 168.3381**					
	**Regression Coefficients**	**Std. Error**	**t**	***p*** **-value**	**−** **95% Conf.** **Lmt**	**+95% Conf.** **Lmt**
Intercept	1144.160	838.0765	1.36522	0.189005	−616.573	2904.893
(1) Type of Mixture (L)	33.324	30.2881	1.10025	0.285722	−30.309	96.957
Type of Mixture (Q)	−2.096	1.0617	−1.97404	0.063926	−4.327	0.135
(2) Type of Binder (L)	−142.485	93.4712	−1.52438	0.144792	−338.861	53.890
Type of Binder (Q)	3.826	2.6484	1.44452	0.165775	−1.738	9.390
1L*2L	0.520	1.3870	0.37504	0.712014	−2.394	3.434
**Effect**	**Variable: PRD_air_; R^2^ = 0.61231; ** $R_{\mathrm{adj}}^{2}$ **= 0.50462; Pure Error MS = 168.3381**					
	**Regression Coefficients**	**Std. Error**	**t**	***p*** **-value**	**−** **95% Conf.** **Lmt**	**+95% Conf.** **Lmt**
Intercept	747.8221	394.9175	1.89362	0.074464	−81.869	1577.513
(1) Type of Mixture (L)	22.0991	14.2723	1.54839	0.138930	−7.886	52.084
Type of Mixture (Q)	0.0549	0.5003	0.10976	0.913810	−0.996	1.106
(2) Type of Binder (L)	−93.2439	44.0454	−2.11699	0.048448	−85.780	−0.708
Type of Binder (Q)	2.8406	1.2480	2.27619	0.035285	0.219	5.463
1L*2L	−1.1778	0.6536	−1.80207	0.088306	−2.551	0.195
**Effect**	**Variable: PRD_air_; R^2^ = 0.61231; ** $R_{\mathrm{adj}}^{2}$ **= 0.50462; Pure Error MS = 168.3381**					
	**Regression Coefficients**	**Std. Error**	**t**	***p*** **-value**	**−** **95% Conf.** **Lmt**	**+95% Conf.** **Lmt**
Intercept	980.219	673.6416	1.45511	0.162859	−435.049	2395.488
(1) Type of Mixture (L)	14.923	24.3454	0.61296	0.547568	−36.225	66.071
Type of Mixture (Q)	−1.730	0.8534	−2.02771	0.057657	−3.523	0.062
(2) Type of Binder (L)	−16.431	75.1317	−1.54969	0.138618	−274.277	41.415
Type of Binder (Q)	2.917	2.1288	1.37043	0.187404	−1.555	7.390
1L*2L	1.328	1.1149	1.19141	0.248965	−1.014	3.671

where: Q—quadratic; L—linear.

## Data Availability

Data available in a publicly accessible repository.
